# Nutrigenomics of High Fat Diet Induced Obesity in Mice Suggests Relationships between Susceptibility to Fatty Liver Disease and the Proteasome

**DOI:** 10.1371/journal.pone.0082825

**Published:** 2013-12-06

**Authors:** Helen Waller-Evans, Christophe Hue, Jane Fearnside, Alice R. Rothwell, Helen E. Lockstone, Sophie Caldérari, Steven P. Wilder, Jean-Baptiste Cazier, James Scott, Dominique Gauguier

**Affiliations:** 1 The Wellcome Trust Centre for Human Genetics, University of Oxford, Oxford, United Kingdom; 2 Cordeliers Research Centre, Institut National de la Santé et de la Recherche Médicale INSERM, UMRS872, University Pierre & Marie Curie, Paris, France; 3 Institute of Cardiometabolism and Nutrition, University Pierre & Marie Curie, Paris, France; 4 Department of Vascular Science, National Heart and Lung Institute, Imperial College London, South Kensington, London, United Kingdom; INRA, France

## Abstract

Nutritional factors play important roles in the etiology of obesity, type 2 diabetes mellitus and their complications through genotype x environment interactions. We have characterised molecular adaptation to high fat diet (HFD) feeding in inbred mouse strains widely used in genetic and physiological studies. We carried out physiological tests, plasma lipid assays, obesity measures, liver histology, hepatic lipid measurements and liver genome-wide gene transcription profiling in C57BL/6J and BALB/c mice fed either a control or a high fat diet. The two strains showed marked susceptibility (C57BL/6J) and relative resistance (BALB/c) to HFD-induced insulin resistance and non alcoholic fatty liver disease (NAFLD). Global gene set enrichment analysis (GSEA) of transcriptome data identified consistent patterns of expression of key genes (*Srebf1*, *Stard4, Pnpla2, Ccnd1*) and molecular pathways in the two strains, which may underlie homeostatic adaptations to dietary fat. Differential regulation of pathways, including the proteasome, the ubiquitin mediated proteolysis and PPAR signalling in fat fed C57BL/6J and BALB/c suggests that altered expression of underlying diet-responsive genes may be involved in contrasting nutrigenomic predisposition and resistance to insulin resistance and NAFLD in these models. Collectively, these data, which further demonstrate the impact of gene x environment interactions on gene expression regulations, contribute to improved knowledge of natural and pathogenic adaptive genomic regulations and molecular mechanisms associated with genetically determined susceptibility and resistance to metabolic diseases.

## Introduction

Genetic predisposition and changes in the environment are associated in the etiology of increasingly prevalent human complex disorders, including the cardiometabolic syndrome (CMS), which combines glucose intolerance, insulin resistance and obesity [1]. Nutritional factors strongly influence CMS onset and progression [2] and may in turn have an impact on associated diseases, either directly or through genotype x environment interactions. Non alcoholic fatty liver disease (NAFLD), which covers conditions ranging from steatohepatitis to cirrhosis [3,4], is the most common liver disease frequently associated with the CMS. With the emergence of genome-wide association studies (GWAS) that have identified genetic factors contributing to these diseases [5,6], functional information on the biological and pathophysiological roles of genes at disease susceptibility loci are crucially needed. High density datasets that gene expression profiling technologies generate can efficiently be used to systematically annotate the function of disease associated loci and to assist the interpretation of GWAS signals.

Genotypes that alter disease susceptibility or resistance induced by nutritional stimuli undoubtedly play an important role in NAFLD and CMS [2]. Metabolic and hormonal responses to dietary changes involve coordinated regulations of complex mechanisms occurring in several organs that maintain glucose and lipid homeostasis [7,8]. Disrupting these adaptive mechanisms, which can be caused by gene x environment interactions, can progressively lead to insulin resistance, obesity and NAFLD. Rodent models provide powerful tools for molecular genetic investigations into the impact of nutritional changes in CMS and NAFLD pathogenesis. High fat diet (HFD) feeding is an efficient system promoting obesity, insulin resistance and NAFLD in the majority of rodent models [9-11]. We have previously shown the breadth of genetically determined physiological and metabolic responses to HFD in inbred mouse strains [12-14]. In particular, mice of the 129S6 strain fed HFD developed liver histopathology resembling NAFLD associated with significantly increased liver triglyceride content and plasma alanine aminotransferase (ALT) and aspartate aminotransferase (AST) levels [13]. In contrast, in strictly identical experimental conditions, HFD-fed BALB/c mice did not show evidence of liver triglyceride accumulation and were devoid of NAFLD, even though levels of ALT and AST were increased in both strains in response to HFD [13], suggesting the involvement of genetically driven mechanisms of resistance to diet induced NAFLD in BALB/c mice.

We report here liver gene expression patterns in mouse strains that show relative resistance (BALB/c) or susceptibility (C57BL/6J, 129S6) to insulin resistance and NAFLD phenotypes in response to prolonged HFD feeding. Results, which highlight biological pathways that account for either natural adaptation to HFD or nutrigenomic predisposition to disease, contribute to improved knowledge of natural and pathogenic adaptive genomic regulations associated with genetically determined susceptibility and resistance to diet-induced insulin resistance and NAFLD. Results from our study complement published transcriptome data in other models of CMS and NAFLD towards a comprehensive picture of perturbed molecular mechanisms that can be involved in the onset and progression of these disorders. 

## Materials and Methods

### Ethics Statement

All experiments in fat fed mice were carried out with permission of UK Home Office personal and project (PPL1995) licences conditions under the Animal [Scientific Procedures] Act 1986 and approved by the local ethical review committee on animal care of the University of Oxford.

### Animals

Male mice from BALB/c and C57BL/6J strains were bred locally using stocks from the Jackson Laboratory. Mice were maintained under standard conditions and fed *ad libitum* a standard carbohydrate diet (CHD) chow (B&K Universal Ltd, Hull, UK). At 5 weeks of age, one group of mice from each strain was transferred to a 40% high fat diet (HFD) (Special Diets Services, Witham, UK) (Table S1), containing 32% lard oil and 8% corn oil, and separate strain and age matched control groups remained on CHD for the duration of the diet trial. 

### Glucose tolerance and insulin secretion tests

Body weight (BW) was measured and intraperitoneal glucose tolerance tests (IPGTT) were performed in anesthetized mice (Sagatal, Rhône Mérieux, Harlow, UK) following an overnight fast at 8, 12, 20 and 28 weeks of age (i.e. after 3, 7, 15 and 23 weeks of HFD feeding) as previously described [12]. A solution of glucose (2g/kg BW) was injected intraperitoneally and blood samples were collected from the tail vein before the injection and 15, 30 and 75 minutes afterward to quantify blood glucose (Accucheck, Roche Diagnostics, Welwyn Garden City, UK) and immunoreactive insulin (IRI) (Mercodia, Uppsala, Sweden). Cumulative glycemia (CumG) and insulinemia (CumIRI) were calculated as the increment of the values of plasma glucose and insulin, respectively, during the IPGTT.

### Tissue sampling

At five months, mice were individually housed in metabolic cages to determine food consumption. Digestible energy was calculated by multiplying the amounts of CHD and HFD eaten by 14 and 22.17, respectively. Following an overnight fast, Blood samples were collected by cardiac puncture and plasma was separated by centrifugation and stored at -80°C for cholesterol assay (ABX diagnostics, Shefford, UK). Epididymal fat pads (EFP) were collected and weighed. Adiposity index (AI) was calculated as the ratio between EFP weight and BW. Liver samples were collected and either fixed in neutral buffered formalin solution (Surgipath Europe Ltd, Peterborough, UK), dehydrated, embedded in paraffin, sectioned at 4 µm and stained with haematoxylin and eosin (H&E) or snap frozen in liquid nitrogen and stored at -80°C for RNA preparation.

### Determination of alanine transaminase (ALT) activity and liver triglycerides content

Liver samples (50mg) from fat fed and control BALB/c and C57BL6/J mice were homogenised in an ALT assay buffer for the determination of ALT activity using a commercial colorimetric assay (Abcam, Paris, France). A separate batch of liver extracts was prepared and incubated in a buffer containing NP40 (5%) and supernatants containing the triglycerides were separated. Triglycerides concentration was determined on the supernatant fraction using a commercial colorimetric assay according to manufacturer's recommendations (Abcam, Paris, France). ALT activity and triglycerides concentration were determined by measuring OD at 570nm.

### Gene transcription profiling

Total RNA form liver of six mice per group was extracted using Trizol reagent (Invitrogen Life Technologies, Paisley, UK) and cleaned with RNeasy columns (Qiagen Ltd., Crawley, UK). RNA concentrations and integrity were assessed using an Agilent 2100 Bioanalyser (Agilent Technologies, Waldbronn, Germany). RNA probes prepared from BALB/c mice were hybridized to Affymetrix expression arrays 430 A and B (Affymetrix UK ltd, High Wycombe, UK), containing 22,690 and 22,576 probesets, respectively, and allowing quantification of the abundance of transcripts corresponding to 13,250 (chip A) and 7577 (chip B) independent gene and EST sequences. Probes prepared from C57BL/6J mice were hybridized to Affymetrix arrays U430 2.0, which were designed to contain all probesets of arrays 430 A and B on a single chip. Experiments were performed according to Affymetrix protocols as previously described [14]. Experiments are MIAME compliant and full protocols and data are publicly available (www.ebi.ac.uk/arrayexpress/) under the accessions E-MTAB-488 (BALB/c) and E-MEXP-1755 (C57BL/6J).

### Statistical analyses

Univariate General Linear Model (GLM) was performed for phenotype analyses using SPSS. To assess differences between the strains fed CHD and HFD, Fisher’s LSD and Tamhane’s T2 post hoc tests were used according to Levene’s test for equality of variance.

Processing and analysis of the Affymetrix .CEL file data was carried out using the BioConductor packages in the R language and environment as previously reported [14]. Gene chip data were normalised by use of RMA quantile normalization [15]. For the BALB/c datasets, the A and B chips were normalised separately. The use of different Affymetrix arrays for BALB/c and C57BL/6J prevented direct interstrain analyses. We used the RMA expression index, ignoring Mismatch values for background correction and LIMMA (Linear Models for MicroArray data, Bioconductor project) to assess significant gene expression differences between groups. To correct for multiple testing, we used the false discovery rate of Benjamini & Hochberg [16] to control the proportion of false positives at 5%.

#### Quantitative real-time PCR

Assays were performed on a Rotor-Gene 3000TM system (Corbett Research, Milton, UK) using the QuantiTect SYBR Green PCR kit (Qiagen Ltd, Crawley, UK). Gene expression was normalised against the expression of GAPDH. Experiments were performed in triplicate with samples prepared from six animals per group. Statistical significance between HFD- and CHD-fed mice was determined using a non parametric Mann Whitney test. Oligonucleotide sequences are given in Table S2.

#### Ubiquitination of PPARG

Total proteins were prepared from liver samples of the BALB/c, C57BL/6J and 129S6 mice fed CHD or HFD used for transcription profiling. The amount of ubiquitinated PPARG was determined by sandwich ELISA, using an immobilized mouse anti-PPARG antibody (Abcam, Cambridge, UK) and a mouse polyubiquitinated antibody (Enzo Life sciences, Villeurbanne, France). Revelation was carried out with a goat anti-mouse (HRP) antibody by addition of 3,35,5Tetramethylbenzidine (Sigma Aldrich, St Quentin, France). Ubiquitinated PPARG levels were quantified by OD reading at 450nm.

### Biological pathway analysis

Gene set enrichment analysis (GSEA) was used to assess biological pathways affected by high fat feeding in each strain [17]. For each strain, Affymetrix probesets were first ranked according to differential expression (t-statistic) between groups fed HFD or CHD. A custom chip file mapping between mouse Affymetrix probeset IDs and human gene symbols was used in conjunction with the most recent version of the Kyoto Encyclopedia of Genes and Genomes (KEGG) pathway gene sets file available from the GSEA website. The ranked lists, gene sets and chip files were then submitted to GSEA and each KEGG pathway was tested for enrichment in each list. Pathways with a false discovery rate (FDR) q-value below 0.05 were considered significant. Normalized Enrichment Scores (NES) were calculated for each gene set. NES reflect the degree to which a gene set is overrepresented at the top or bottom of a ranked list of genes created by GSEA for each gene set according to differential gene expression between mice fed HFD or CHD. Positive or negative NES indicate that the gene set is overexpressed or underexpressed, respectively.

## Results

### Pathophysiological features of adaptation to fat feeding

**Figure 1:**
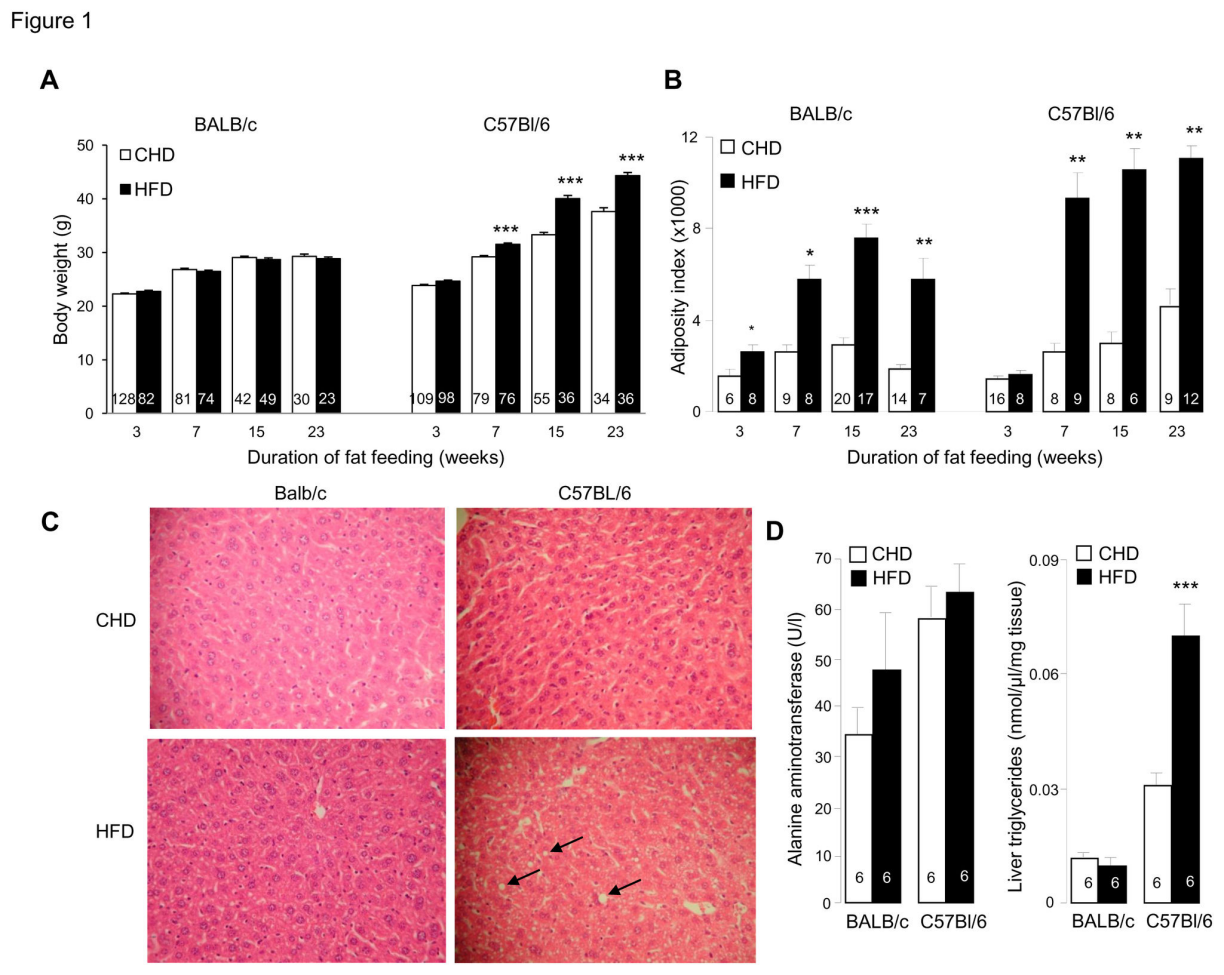
Effects of prolonged high fat diet (HFD) feeding on obesity and fatty liver variables. Body weight (A), adipose tissue weight (B), liver histopathology (magnification 20X) (C) and liver ALT activity and triglyceride concentration (D) were analysed in C57BL/6J and BALB/c fed HFD or control diet. Data are shown as means±SEM. Number of mice used is shown in the histograms. *p<0.05, **p<0.01, ***p<0.001 significantly different between HFD fed mice and age matched CHD fed controls.

**Figure 2:**
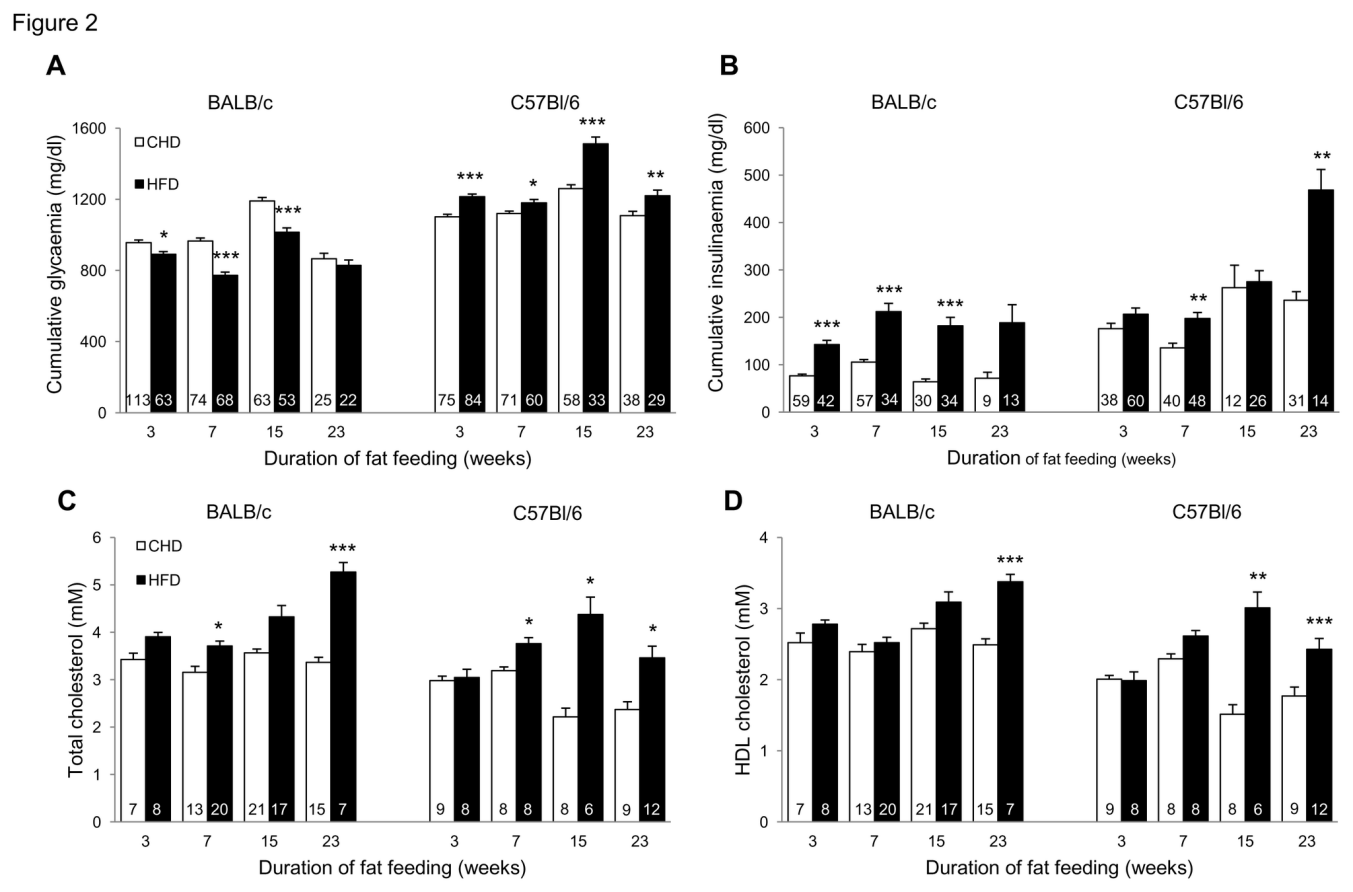
Effects of prolonged high fat diet (HFD) feeding on glucose and lipid regulations. Glucose tolerance (A), insulin secretion (B) and total and HDL cholesterol (C,D) were analysed in C57BL/6J and BALB/c fed HFD or control diet. Values are expressed as means±SEM. Number of mice used is shown in the histograms. *p<0.05, **p<0.01, ***p<0.001, significantly different to age-matched CHD fed mice of the same strain.

In response to HFD, C57BL/6J mice exhibited increased body weight and adiposity index, whereas in BALB/c mice body weight remained generally unchanged despite increased adiposity (Figure **1**), suggesting that lean body mass is lower in this strain and/or that the much lower raise in adiposity index in BALB/c than in C57BL/6J does not translate in noticeable increase in body weight in BALB/c. Digestible energy intake was not significantly affected by HFD in either strain (data not shown). Fat feeding induced marked and persistent enhanced insulin secretion in both strains, which was associated with impaired glucose homeostasis in C57BL/6J and paradoxically improved glucose tolerance in BALB/c (Figure **2A,B**). Plasma total and HDL cholesterol concentrations were generally increased by HFD feeding in both strains (Figure **2C,D**).

To investigate the impact of HFD feeding on liver damage phenotypes relevant to NAFLD, liver histological analysis was carried out in BALB/c and C57BL/6J mice, at a stage (15 weeks of HFD feeding) when alterations in physiological phenotypes are well-established in both strains. Mice of the BALB/c and C57BL/6J strains fed CHD exhibited normal liver histology (Figure **1C**). In response to HFD, liver histology remained unchanged in BALB/c, whereas evidence of fatty liver was observed in C57BL/6J mice (Figure **1C**). ALT activity was increased in response to HFD in both BALB/c and C57BL/6J mice, but differences were not statistically significant (Figure **1D**). Similar concentration of liver triglycerides in BALB/c mice fed either CHD or HFD confirmed resistance to HFD-induced fatty liver disease in BALB/c (Figure **1D**). In contrast in C57BL/6J mice, prolonged HFD feeding was associated with a significant increase in liver triglycerides content (Figure **1D**), consistent with liver histopathological features observed in mice of this strain when fed HFD.

Overall these data show that two mouse strains maintained in strictly identical experimental conditions adapt to the same nutritional stimulus with phenotypic features that are conserved (eg. increased adiposity) or divergent (insulin sensitivity, NAFLD), supporting the strong impact of gene x environment interactions in strain-specific phenotypic adaptations to dietary changes.

### Overview of genome-wide gene expression profiles

To assess at the molecular level the consequences of HFD on liver biology in C57BL/6J and BALB/c mice, liver transcriptomes were generated. The global effect of HFD on statistically significant transcriptional changes was more pronounced in C57BL/6J (1878 probesets for 1494 different genes) than in BALB/c (489 probesets for 431 different genes) (Table S3). A total of 118 genes were found differentially expressed in response to fat feeding in both strains, and only a minority (13) showed inconsistent directional change of expression. Both these and genes showing strain specific expression patterns in response to HFD, might underlie genetic predisposition of C57BL/6J to steatohepatitis and, at least partly, insulin resistance.

### Gene expression responses to HFD feeding

We initially investigated differential expression of individual genes in the liver transcriptomes that may be involved in natural and pathological adaptations to HFD feeding in BALB/c and C57BL/6J. When the effect of HFD on transcription ratios was considered, the magnitude of the effects for the top ranking genes was stronger in C57BL/6J (from -24.9 to +8.3) than in BALB/c (from -4.8 to +3.6) (Table S3). The top ranking genes in BALB/c (downregulation of *Cyp3a11*, the monooxygenase *Moxd1* and the 3-hydroxyacyl Coenzyme A Ehhadh, and upregulation of the carbonic anhydrase *Car3*), often showed identical patterns of adaptation to HFD in C57BL/6J. In contrast, genes showing the strongest magnitude of transcriptional response to HFD in C57BL/6J were specific to this strain and included downregulated expression of genes encoding metallothioneins (*Mt1*, *Mt2*), insulin-like growth factor binding protein 1 (*Igfbp1*), aspartate aminotransferase (*Got1*) and glucose-6-phosphatase (*G6pc*), and upregulation expression of glucokinase (*Gck*) and adipsin (*Adn*).

We then analysed coordinated expression of genes involved in similar biological processes relevant to insulin resistance and NAFLD. In BALB/c fat feeding stimulates the expression of genes concerned with lipoprotein uptake (*Srebf1*, *Stard4*, *Ldlr*) and fatty acid and cholesterol synthesis (*Hmgcs1*, *Hmgcr*, *Fdps*, *Fdft1*, *Acly*, *Fasn*), and down regulates genes involved in lipoprotein secretion (*ApoA4*, *ApoA5*, *Mttp*), fatty acid uptake and elongation (*Elovl3, Pnpla2*), intracellular transport of cholesterol (*Npc1*), mitochondrial transfer of acyl CoA (*Cpt2*, *Crot*, *Crat*, *Slc25a20*) and beta oxidation (*Acox1*, *Acox2*, *Acadm*, *Acadvl*, *Hadhb*, *Ech1*, *Acaa1*) (Table S3). In C57BL/6J, genes involved in lipoprotein secretion and lipid efflux (*ApoA4*, *ApoA5*, *Abcd2*, *Saa2*), fatty acid transport, synthesis, modification and elongation (*Fabp1*, *Fabp2, Srebf1*, *Stard4*, *Elovl5*, *Sc5d*) and hepatocyte growth and proliferation (*Catnb*, *Cav1*, *Cav2*) were up regulated by HFD.

These results underline the complexity of gene transcription adaptation to HFD in BALB/c and C57BL/6J, combining strain specific and conserved mechanisms. They indicate that the strongest transcriptional impact of HFD in C57BL/6J concerns genes differentially expressed specifically in this strain, whereas genes showing the highest magnitude of transcription changes in BALB/c also exhibit similar response to HFD in C57BL/6J. These data also suggest a possible role of coordinated expression of SREBF1 and STARD4 in normal adaptation to HFD feeding.

### Pathway analysis of liver transcriptomes

**Figure 3:**
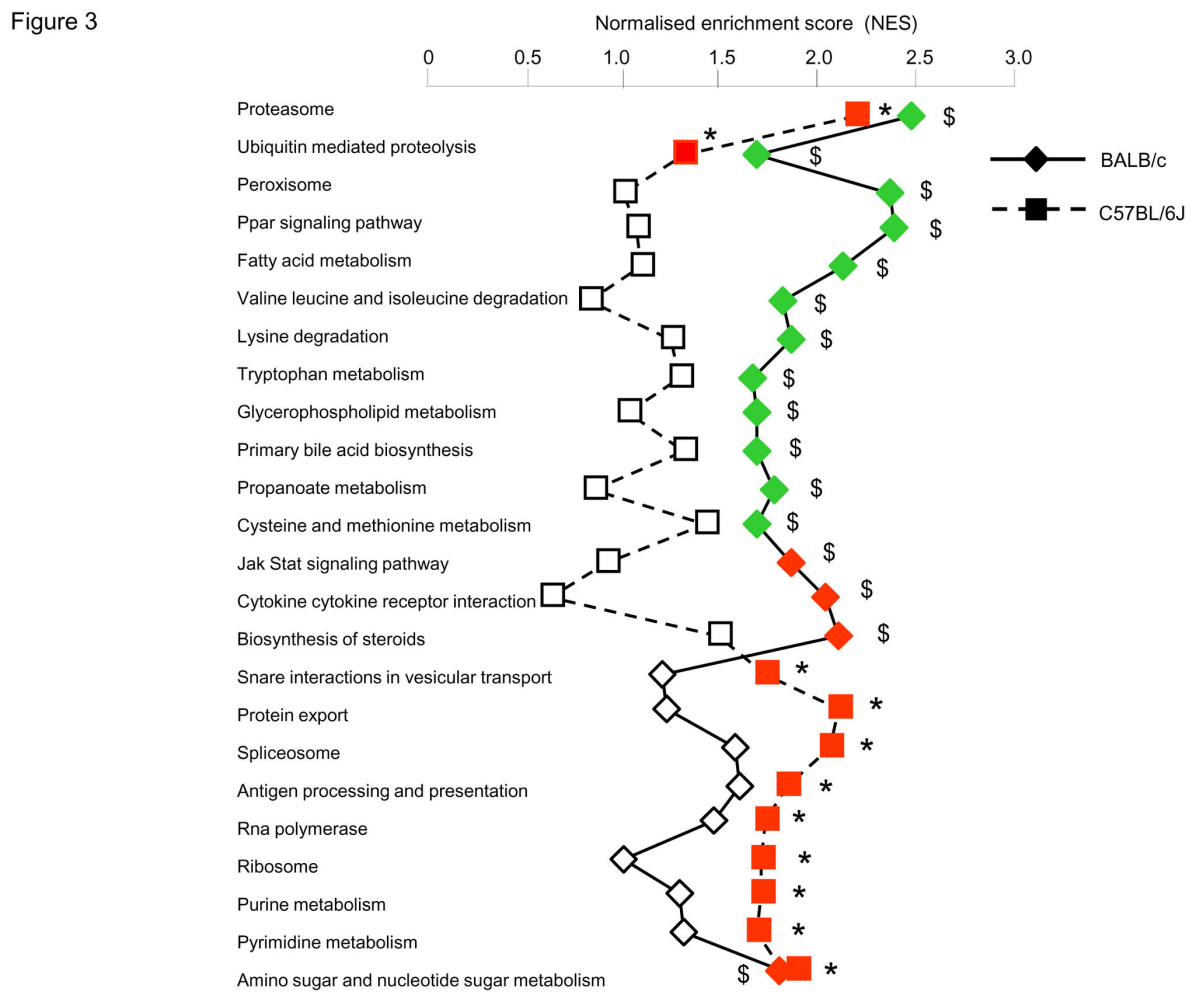
Overview of HFD-induced liver gene transcription changes identified by GSEA in C57BL/6J and BALB/c mice. Only KEGG biological pathways significantly upregulated (red) or downregulated (green) by HFD in at least one of the two strains are reported. The (absolute) Normalised Enrichment Score (NES) computed by GSEA is plotted (with larger values indicative of significant enrichment). FDR q-values (<0.05) were used to identify statistically significant effects of fat feeding on the pathways in C57BL/6J (*) and BALB/c ($).Genes contributing to pathway enrichment are listed in Table 1.

**Figure 4:**
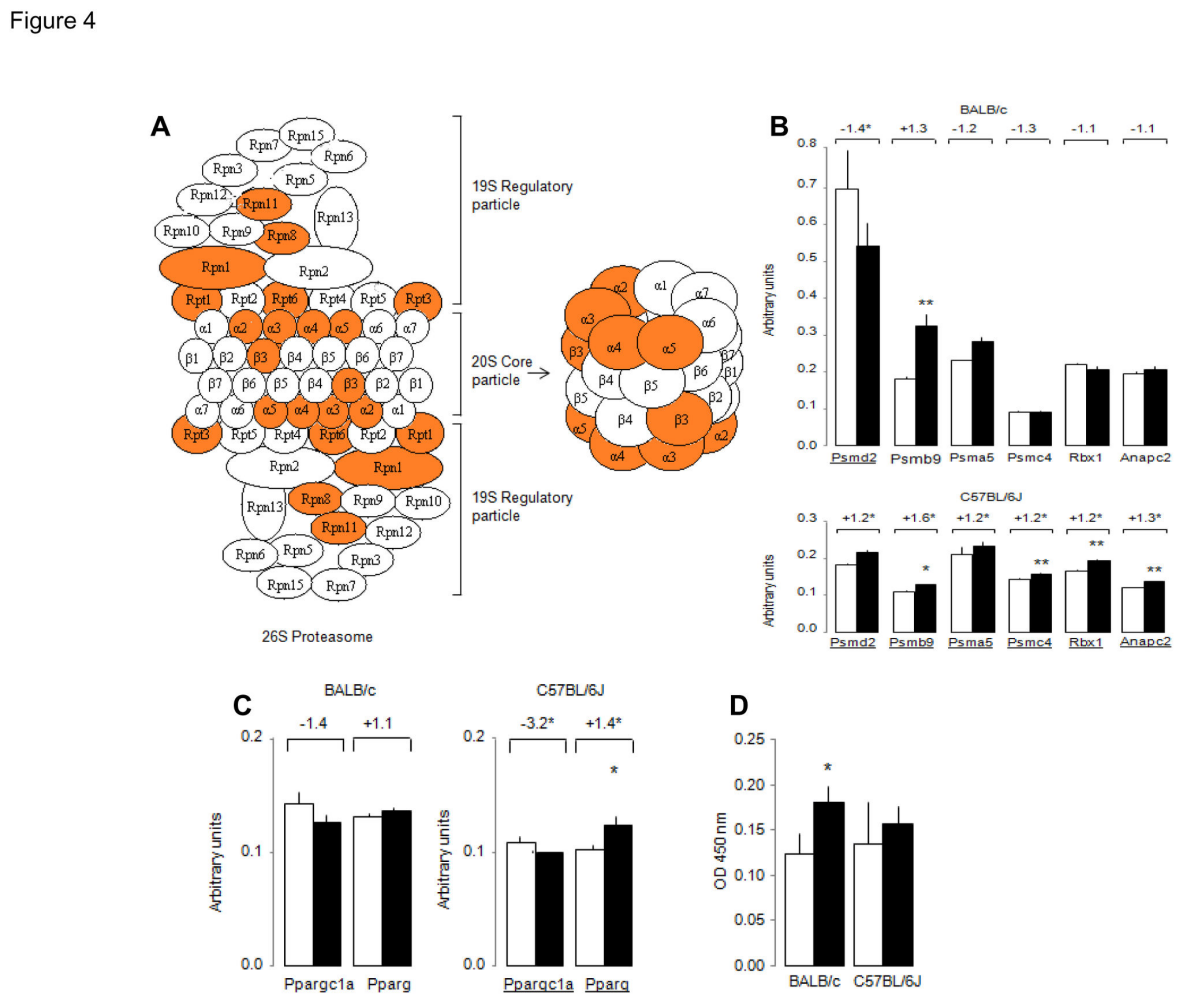
Validation of liver transcriptome pathway analyses. Protein elements of the proteasome pathway encoded by genes significantly (P<0.05) upregulated by HFD in C57BL/6J mice are highlighted in orange (A) (from www.kegg.jp/kegg). QRT-PCR-based transcription of genes in the proteasome (B) and PPAR (C) pathways and ubiquitin binding to PPARG (D) were assessed with liver samples from mice fed HFD (■) or CHD (*) used for transcriptome and phenotype studies (Figs. 1 and 2). Affymetrix-based transcription ratios in response to HFD feeding are reported above the QRT-PCR data and differential expressed genes on the Affymetrix datasets are underlined. Details of genes and oligonucleotides used for QRT-PCR are given in Table 1 and Table S2, respectively. Data are means±SE. *p<0.05, **p<0.01 significantly different between HFD fed mice and controls.

To identify global gene transcription patterns associated with diet-induced resistance (BALB/c) and susceptibility (C57BL/6J) to NAFLD, we applied GSEA which allowed exploration of changes in gene pathways (Figure **3**) and identification of individual genes driving altered pathway expression (Table 1). In both strains adaptation to HFD feeding involved consistent activation of the metabolism of amino and nucleotide sugars, which may reflect adaptive mechanisms unrelated to disease pathogenesis. In contrast, fat feeding induced opposite transcriptional regulation patterns of the proteasome pathway in the two strains (Figure **3**), through significant transcription down-regulation of *Psma5*, *Psmb4*, *Psmd2*, *Psmd11* in BALB/c and up-regulation of *Psma2*, *Psma4*, *Psma5*, *Psma7*, *Psmb3*, *Psmc2* in C57BL/6J (Table 1, Figure **4A**). Expression of the ubiquitin mediated proteolysis pathway was also significantly downregulated by HFD in BALB/c (NES=-1.685, P=0.037) and strongly but not statistically upregulated in C57BL/6J (NES=1.308, P=0.41) (Figure **3**). Of note, HFD induced statistically significant upregulation of both ubiquitin C and a series of ubiquitin regulatory genes specifically in C57BL/6J (Table 1).

**Table 1:** Effects of fat feeding on hepatic transcription of genes contributing to changes in KEGG pathways conserved or divergent in BALB/c and C57BL/6J mice.

**Pathway/Gene**		**C57BL/6J**		**BALB/c**	
**Symbol**	**Gene description**	**TR**	**P**	**TR**	**P**
**Proteasome**					
*Psma2* (α2)	Proteasome subunit, alpha type 2	1.3	0.004	-1.2	Ns
*Psma4* (α3)	Proteasome subunit, alpha type 4	1.2	0.03	-1.1	Ns
*Psma5* (α5)	Proteasome subunit, alpha type 5	1.2	0.026	-1.2	0.05
*Psma7* (α4)	Proteasome subunit, alpha type 7	1.2	0.045	-1.2	Ns
*Psmb3* (β3)	Proteasome subunit, beta type 3	1.2	0.035	-1.1	Ns
*Psmb4* (β7)	Proteasome subunit, beta type 4	1.1	Ns	-1.3	0.01
*Psmb9*	Proteasome subunit, beta type 9	1.6	0.034	1.3	Ns
*Psmc2* (Rpt1)	Proteasome 26S subunit, ATPase 2	1.2	0.044	-1.1	Ns
*Psmc4* (Rpt3)	Proteasome 26S subunit, ATPase 4	1.2	0.02	-1.3	Ns
*Psmc5* (Rpt6)	Proteasome 26S subunit, ATPase 5	1.2	0.036	-1.2	Ns
*Psmd2* (Rpn1)	Proteasome 26S subunit, non-ATPase, 2	1.2	0.043	-1.4	0.019
*Psmd4* (Rpn10)	Proteasome 26S subunit, non-ATPase, 4	1.1	Ns	-1.3	0.014
*Psmd7* (Rpn8)	Proteasome 26S subunit, non-ATPase, 7	1.2	0.013	1	Ns
*Psmd11* (Rpn6)	Proteasome 26S subunit, non-ATPase, 11	1.1	Ns	-1.3	0.021
*Psmd14* (Rpn14)	Proteasome 26S subunit, non-ATPase, 14	1.3	0.012	-1.1	Ns
*Psme4*	Proteasome activator subunit 4	1.2	0.049	-1.2	Ns
**Ubiquitin-mediated proteolysis**					
*Anapc2*	Anaphase promoting complex subunit 2	1.3	0.004	-1.1	Ns
*Anapc10*	Anaphase promoting complex subunit 10	1.3	0.038	-1.1	Ns
*Rbx1*	Ring-box 1	1.2	0.026	-1.1	Ns
*Ube2d1*	Ubiquitin-conjugating enzyme E2D 1	1.3	0.041	-1.2	Ns
*Ube2d2*	Ubiquitin-conjugating enzyme E2D 2	1.2	0.033	1.1	Ns
*Ube2d3*	Ubiquitin-conjugating enzyme E2D 3	1.4	0.009	-1.1	Ns
*Ube2e2*	Ubiquitin-conjugating enzyme E2E 1	1.4	0.03	-1.1	Ns
**PPAR signalling**					
*Acox2*	Acyl-Coenzyme A oxidase 2, branched chain	-1.2	Ns	-1.3	0.05
*Angptl4*	Angiopoietin-like 4	-1.9	0.003	-2.7	<0.001
*Apoa5*	Apolipoprotein A-V	-1.5	0.029	-1.5	0.011
*Cyp8b1*	Cytochrome P450, family 8, subfamily b	-1.1	Ns	-1.6	0.016
*Dbi*	Diazepam binding inhibitor	1.1	Ns	-1.2	0.016
*Fabp1*	Fatty acid binding protein 1, liver	1.3	0.032	-1.2	Ns
*Fabp2*	Fatty acid binding protein 2, intestinal	1.4	0.007	-1.2	Ns
*Hmgcs2*	3-hydroxy-3-methylglutaryl-Coenzyme A synthase 2	1.2	Ns	-1.4	0.035
*Pck1*	Phosphoenolpyruvate carboxykinase 1, cytosolic	-1.6	0.007	-2.2	<0.001
*Pdpk1*	3-phosphoinositide dependent protein kinase-1	-1.3	0.045	-1	Ns
*Rxrα*	Retinoid X receptor alpha	1.3	0.044	-1.1	Ns
*Sorbs1*	Sorbin and SH3 domain containing 1	2.2	<0.001	-1.7	Ns
*Ubc*	Ubiquitin C	1.3	0.011	-1.4	Ns
**PPAR signalling, Fatty acid metabolism**					
*Acadm*	Acetyl-Coenzyme A dehydrogenase, medium chain	1.1	Ns	-1.3	0.02
*Acox1*	Acyl-Coenzyme A oxidase 1, palmitoyl	-1.1	Ns	-1.4	0.035
*Cpt2*	Carnitine palmitoyltransferase 2	-1.1	Ns	-1.4	0.002
*Ehhadh*	Enoyl-Coenzyme A, hydratase	-1.5	0.039	-2.9	<0.001
**Fatty acid metabolism**					
*Acadvl*	Acyl-Coenzyme A dehydrogenase, very long chain	-1	Ns	-1.3	0.017
*Acat1*	Acetyl-Coenzyme A acetyltransferase 1	1.2	0.02	-1.3	0.049
*Acat2*	Acetyl-Coenzyme A acetyltransferase 2	1	Ns	1.4	0.019
*Adh4*	Alcohol dehydrogenase 4 (class II), pi polypeptide	2.2	<0.001	1.3	Ns
*Dci*	Dodecenoyl-Coenzyme A delta isomerase	1	Ns	-1.3	0.012
*Hadhb*	Hydroxyacyl-Coenzyme A dehydrogenase	1	Ns	-1.4	0.009
*Hsd17b4*	Hydroxysteroid (17-beta) dehydrogenase 4	1	Ns	-1.3	0.013
**Jak-Stat signalling**					
*Ccnd1*	Cyclin D1	1.7	0.001	1.7	0.006
*Ccnd2*	Cyclin D2	1.4	0.046	1.3	0.032
*Ghr*	Growth hormone receptor	1.4	0.031	1.6	0.032
*Ifnar1*	Interferon (alpha and beta) receptor 1	1.2	0.005	1.2	Ns
*Il2rb*	Interleukin 2 receptor, beta chain	-1	Ns	1.3	0.05
*Lifr*	Leukemia inhibitory factor receptor	1	Ns	1.3	0.013
*Pik3r1*	Phosphatidylinositol 3-kinase, regulatory subunit	1.7	0.005	1.1	Ns
*Prlr*	Prolactin receptor	1.5	Ns	1.4	0.004
*Stat5b*	Signal transducer and activator of transcription 5B	-1.3	0.028	-1	Ns
**Protein_export**					
*Hspa5*	Heat shock 70kD protein 5	3.2	<0.001	1.8	Ns
*Sec61a1*	Sec61 alpha 1 subunit	1.5	0.009	1.1	Ns
*Sec61b*	Sec61 beta subunit	1.7	0.003	1.1	Ns
*Sec63*	SEC63-like (S. cerevisiae)	1.5	0.007	1.1	Ns
*Spcs2*	Signal peptidase complex subunit 2	1.5	0.019	1.3	Ns
*Spcs3*	Signal peptidase complex subunit 3	1.5	0.001	1.2	Ns
*Srp9*	Signal recognition particle 9	1.2	0.01	1	Ns
*Srp14*	Signal recognition particle 14	1.3	0.019	-1.2	Ns
*Srp19*	Signal recognition particle 19	1.3	0.007	1.1	Ns
*Srp68*	Signal recognition particle 68	1.3	0.008	1.1	Ns
*Srprb*	Signal recognition particle receptor, B subunit	1.4	0.02	1.5	Ns
**Spliceosome**					
*Hspa1a*	Heat shock protein 1A	1.8	<0.001	1.1	Ns
*Hspa1b*	Heat shock protein 1B	4	0.033	1.7	Ns
*Hspa8*	Heat shock protein 8	2.6	0.01	1	Ns
*Lsm6*	LSM6 homolog, U6 small nuclear RNA associated	1.4	0.015	-1.1	Ns
*Pcbp1*	Poly(rC) binding protein 1	1.2	0.008	1.1	Ns
*Phf5a*	PHD finger protein 5A	1.3	0.012	-1	Ns
*Prpf3*	PRP3 pre-mRNA processing factor 3 homolog (yeast)	1.3	0.01	1.1	Ns
*Sf3a3*	Splicing factor 3a, subunit 3	1.3	0.013	1.1	Ns
*Sf3b5*	Splicing factor 3b, subunit 5	1.3	0.006	-1	Ns
*Sfrs2*	Splicing factor, arginine/serine-rich 2 (SC-35)	1.5	0.001	1.2	Ns
*Sfrs3*	Splicing factor, arginine/serine-rich 3 (SRp20)	1.8	<0.001	1	Ns
*Sfrs7*	Splicing factor, arginine/serine-rich 7	1.6	<0.001	1.2	Ns
*Snrpa*	Small nuclear ribonucleoprotein polypeptide A	1.3	0.006	1.2	0.013
*Snrpd1*	Small nuclear ribonucleoprotein D1	1.3	0.031	1.2	Ns
*Snrpe*	Small nuclear ribonucleoprotein E	1.2	0.041	1	Ns
*Thoc4*	THO complex 4	1.5	0.009	-1.1	Ns
*Usp39*	Ubiquitin specific protease 39	1.3	0.03	1.1	Ns
**Steroid biosynthesis**					
*Fdft1*	Farnesyl diphosphate farnesyl transferase 1	2.1	0.013	2	0.008
*Fdps*	Farnesyl diphosphate synthetase	1.8	Ns	2.5	0.029
*Ggps1*	Geranylgeranyl diphosphate synthase 1	1	Ns	1.3	0.015
*Hmgcr*	3-hydroxy-3-methylglutaryl-Coenzyme A reductase	1.2	Ns	1.9	0.024
*Idi1*	Isopentenyl-diphosphate delta isomerase	1.5	Ns	2	0.018
*Lss*	Lanosterol synthase	1.2	0.029	1.6	0.002
*Mvd*	Mevalonate (diphospho) decarboxylase	1.2	Ns	1.7	0.007
*Nsdhl*	NAD(P) dependent steroid dehydrogenase-like	1.6	Ns	1.4	0.035
*Sc4mol*	Sterol-C4-methyl oxidase-like	2.4	Ns	2.6	0.058
*Sqle*	Squalene epoxidase	2.8	Ns	2.1	0.031

Only genes in these pathways showing statistically significant (P<0.05) differential expression in response to HFD in at least one strain are reported. Transcription ratio (TR) of genes showing statistically significant downregulation in response to HFD is highlighted in green and TR of significantly upregulated genes is highlighted in red. Ns, not statistically significant. Additional information (Entrez Gene and Affymetrix probeset ID) are in Table S3.

Specifically in BALB/c mice, fat feeding induced significant expression downregulation of PPAR signalling (NES=-2.378, P<0.001), peroxisome (NES=-2.360, P<0.001), metabolism of fatty acids and amino acids (NES=-2.117, P<0.001), and upregulation of cytokine cytokine receptor interaction (NES=2.043, P<0.001), biosynthesis of steroids (NES=2.098, P<0.001) and JAK STAT signalling (NES=1.864, P=0.017) (Figure **3**, Table 1). Even though several genes in the JAK STAT pathway were consistently upregulated by HFD in both strains (Table 1), the vast majority of genes in this pathway were upregulated in BALB/c, whereas an equivalent number of genes were up and down regulated in C57BL/6J (Figure S1), resulting in non significant NES (P=0.82) in C57BL/6J (Figure **3**). Upregulated expression of protein export (NES=2.106, P<0.001), spliceosome (NES=2.054, P<0.001) and Snare interactions in vesicular transport (NES=1.740, P=0.028) was specific to the response of C57BL/6J mice to HFD (Figure **3**, Table 1), suggesting activation of cell regeneration processes. Even though the PPAR signalling pathway was not globally affected by HFD in C57BL/6J mice (NES=1.072, P=0.64), expression of key genes in this pathway (*Pparg*, *Ppargc1a*, *Rxrα*) was significantly altered (Table S3).

These strain-specific alterations in diet-reactive gene expression patterns may directly reflect susceptibility and resistance to genetically determined NAFLD and point to a pivotal role of increased proteasome activity on NAFLD.

#### Validation of altered gene and pathway expression regulation

To test accuracy of Affymetrix-based gene expression data, we performed qRT-PCR analysis of selected genes of the proteasome (Figure **4A**), ubiquitin-mediated proteolysis and PPAR signalling pathways. Significant stimulation of liver expression of *Psmb9*, *Psmc4*, *Rbx1*, *Anapc2* and *Pparg* by HFD in C57BL/6J mice and generally unaffected expression of these genes in BALB/c provide support to Affymetrix array results (Figures **4B,C**). To investigate the biological consequences of differential regulation of ubiquitin mediated proteolysis by HFD in these mice, we quantified liver ubiquitination of PPARG, which requires ubiquitin-mediated proteolysis for activation. We show that ubiquitin binding to PPARG is increased by HFD specifically in BALB/c in the absence of PPARG transcription changes (Figure **4D**). These results suggest that differential regulation of liver proteasome transcription and PPARG expression and ubiquitination may at least partly account for resistance (BALB/c) and susceptibility (C57BL/6J) to HFD-induced NAFLD.

## Discussion

We report conserved and divergent HFD-induced liver gene expression in mouse strains, which underlie physiological responses to the dietary stimulus and genetically-determined increased susceptibility (C57BL/6J) or relative resistance (BALB/c) to liver histopathology resembling NAFLD. Analyses of the expression of both individual genes and biological pathways point to molecular mechanisms contributing to NAFLD and underline the etiological role of gene x environment interactions in the disease. 

In strictly identical experimental conditions, HFD-fed C57BL/6J and BALB/c developed concordant pathophysiological features (hypercholesterolemia, enhanced insulin secretion, increased adiposity), as well as contrasting phenotypic adaptations to HFD in relation to the regulation of glucose tolerance, body weight and liver histology and triglycerides content. Obesity, insulin resistance and NAFLD are hallmarks of adaptation to HFD feeding in many inbred mouse strains [18,19]. C57BL/6J mice exhibited pathophysiological responses to fat feeding similar to 129S6 mice previously investigated using a strictly identical experimental design [14], and reduced severity of liver histopathology in C57BL/6J when compared to 129 mice has also been reported [11]. Our observation of NAFLD resistance in BALB/c mice [13] has recently been confirmed [11] and was explained by reduced fatty acid uptake [11], which concurs with our gene expression data. BALB/c is therefore a particularly important model to identify molecular mechanisms underlying NAFLD resistance.

Systematic application of identical experimental protocols allowed comparisons of liver transcriptomes derived from NAFLD susceptible (C57BL/6J, 129S6) and resistant (BALB/c) strains to identify genes associated with NAFLD and liver insulin resistance. Analysis of genes showing the strongest expression changes in response to HFD pointed to the sharp downregulation of transcription of *Igfbp1*, *G6pc* and metallothioneins (*Mt1*, *Mt2*), which may contribute to liver anomalies in C57BL/6J. Altered liver expression of IGFBP1 and G6P has been reported in patients with NASH [20,21] and metallothioneins may play preventive roles against obesity through reduction of oxidative stress. Reduced liver expression of both *Mt1* and *Mt2* is associated with glucose intolerance and liver steatosis in Nagoya-Shibata-Yasuda (NSY) mice [22] and metallothionein inactivation in mice results in obesity and increased accumulation of fat in the liver when mice are fed HFD [23].

Further analysis of individual genes of biological relevance to NAFLD in HFD-fed C57BL/6J highlighted the coordinated transcription upregulation of beta catenin (*Catnb*) and caveolins (*Cav1*, *Cav2*) which have been associated with steatohepatitis [24,25] and may underlie mechanisms compensating liver structural alterations. An important finding was the systematic stimulation of *Srebf1* transcription by HFD in BALB/c and both NAFLD susceptible strains (Table S4). SREBF1 is a transcription factor playing a central role in hepatic lipid and glucose metabolism, which simulates lipogenic enzymes upon activation by glucose and insulin [26]. HFD-induced hyperinsulinemia in BALB/c, C57BL/6J and 129S6 mice may explain *Srebf1* upregulated transcription. Increased hepatic *Srebf1* expression in these models, which accords with hepatic *Srebf1* expression patterns in fat fed mice [19,27] and in obese Lep^ob/ob^ mice [28], may therefore represent a universal adaptive response to HFD or obesity independent of NAFLD susceptibility.

Beyond analyses of expression of individual genes, the use of a single genome-wide gene expression platform, interrogating transcript abundance for the same genes, and identical analytical methods allowed higher level GSEA-based pathway analysis and identification of biological mechanisms associated with NAFLD susceptibility or resistance in BALB/c, C57BL/6J and 129S6 mice. Consistent upregulated expression of the spliceosome and protein export pathways in both C57BL/6J and 129S6 (Table S4, Figure S2) may indicate the activation of hepatic regeneration processes reactive to liver injury and the stimulation of protein synthesis to compensate upregulation of proteasomal-mediated protein degradation. In contrast, BALB/c-specific upregulated expression of pathways related to JAK STAT signalling and cytokine cytokine receptor interaction (Table S4, Figure S2), which contribute to insulin resistance and steatohepatitis [29,30], suggests the presence of inflammation in HFD-fed BALB/c that does not translate into apparent liver histopathology and may be explained by reduced corticosensitivity in BALB/c [31]. Along the same line, fatty acid metabolism, PPAR signalling and peroxisome showed opposite trends of expression regulation in 129S6 and BALB/c (Figure S2). The nuclear receptors PPARs are key transcriptional regulators of glucose and lipid metabolism [32]. PPARG activation promotes obesity, despite improved insulin sensitivity in liver and adipose tissue [33] and it is generally overexpressed in steatotic liver [34]. HFD-induced downregulation of the PPAR signalling pathway specifically in BALB/c is consistent with resistance to NAFLD and suggests that improved insulin sensitivity in this strain can prevent NAFLD whilst promoting increased adiposity.

Differential regulation of the proteasome and ubiquitin mediated proteolysis pathways in BALB/c, C57BL/6J and 129S6 (Figure S2) is the most striking example of contrasting transcriptional adaptation to HFD in NAFLD-prone and resistant strains. We found that expression of several genes encoding proteins that form the 26S proteasome and participate in the ubiquitin proteasome system (UPS) was consistently upregulated by HFD in C57BL/6J and 129S6, but was downregulated in BALB/c (Table S4). The proteasome is a protein degradation system, which regulates cell differentiation, signal transduction and inflammation. The UPS modifies proteins by linkage of polyubiquitin chains for their subsequent elimination by the proteasome, but also regulates cell proliferation, growth and apoptosis [35]. Enhanced ubiquitination and proteasomal degradation of insulin signalling proteins cause insulin resistance in mouse liver [36] and inhibition of the proteasome is associated with increased insulin secretion [37]. Association between changes in proteasomal activity and obesity and hepatic steatosis was also suggested in transgenic mice [38]. HFD-induced downregulation of both proteasome and ubiquitin-mediated proteolysis pathways in BALB/c may therefore explain both NAFLD resistance and enhanced insulin secretion in this strain.

Altered regulation of proteasome and UPS pathways may have strong repercussions on important cellular systems, including PPAR signalling. PPARG activation is associated with upregulated expression of genes involved in protein ubiquitination [39] and proteasome and UPS elements control the degradation and activity of nuclear receptors, including PPARs and RXRα, to ensure acute transcription regulation [40]. The role of the proteasome on insulin signalling has been demonstrated for PPARG and RXRα [40], which are upregulated by HFD in C57BL/6J. Other differentially expressed genes in HFD-fed C57BL/6J mice that are influenced by proteasome-mediated mechanisms promoted by PPARG activation, include *Catnb*, *Ccnd1* and a cyclin-dependent kinase inhibitor (*Cdkn1b*), which are involved in the regulation of cell proliferation and cell cycle progression, and a glycogen synthase kinase (*Gsk3b*), which regulates CATNB degradation by PPARG [41]. In addition, activation of proteasome and ubiquitin pathways in liver in response to HFD may have consequences not necessarily captured by transcriptome analyses. This includes for example the regulation of SIRT1, a protein known to activate PPARG and SREBF1, which contributes to hepatic steatosis in obesity upon ubiquitination and proteasomal stimulation of its degradation [42]. Our results suggest that coordinated upregulation of the expression of UPS/proteasome and PPAR signalling pathways in response to fat feeding in 129S6 and C57BL/6J mice, and its dissociation in BALB/c, may be central to NAFLD susceptibility and resistance. Increased ubiquitination of PPARG in fat-fed BALB/c mice may represent an adaptive mechanism in this strain to enhance PPARG degradation and prevent NAFLD development.

In conclusion, we show here liver gene transcriptional signatures of natural and pathological adaptations to HFD that determine NAFLD outcomes in a typical gene x environment interactions paradigm in mice. Pathophysiological and molecular features characterise biological adaptations to saturated fat feeding, but may also underlie consequences of differential adaptation of mouse strains to prolonged fasting and differences in nutrients other than fat in the control and high fat diets. Combined with results from the growing number of genome-wide gene expression studies carried out with other mouse models of spontaneous or experimentally-induced obesity and NAFLD, our data contribute to improved knowledge of molecular mechanisms reactive to nutritional challenges and associated with these pathologies. Collectively, integrated analysis of these datasets can provide an overview of altered gene and pathway regulations associated with similar disease conditions to identify key molecular targets for disease treatment in humans.

## Supporting Information

Table S1 **Composition of the high fat diet used to feed C57BL/6J and BALB/c mice.**
(DOCX)

Table S2 **Oligonucleotides designed for quantitative RT-PCR analysis of gene transcription regulation in C57BL/6J and BALB/c mice.**
(DOCX)

Table S3 **Effects of high fat diet feeding on hepatic genome-wide gene transcription regulation.**
(XLSX)

Table S4 **GSEA-based analysis of KEGG biological pathways significantly affected by high fat diet feeding in BALB/c, C57BL/6J and 129S6 mice.**
(XLSX)

Figure S1 **GSEA-derived enrichment plots for the JAK-STAT signalling pathway in BALB/c and C57BL6/J mice.**
(PDF)

Figure S2 **Pathway analysis of the effects of high fat diet on liver gene transcription in C57BL/6J and 129S6 mice.**
(PDF)
