# Supplementary material for: Nutrigenomics of High Fat Diet Induced Obesity in Mice Suggests Relationships between Susceptibility to Fatty Liver Disease and the Proteasome
Source: PLoS One. 2013 Dec 6;8(12):e82825. doi: 10.1371/journal.pone.0082825 (PMC3855786; doi:10.1371/journal.pone.0082825)

**Table S 1.** Composition of the high fat diet (HFD) used to feed ad libitum C57BL/6J and BALB/c mice from 5 weeks of age onwards. Food was manufactured commercially by Special Diets Services (SDS, Witham, UK).


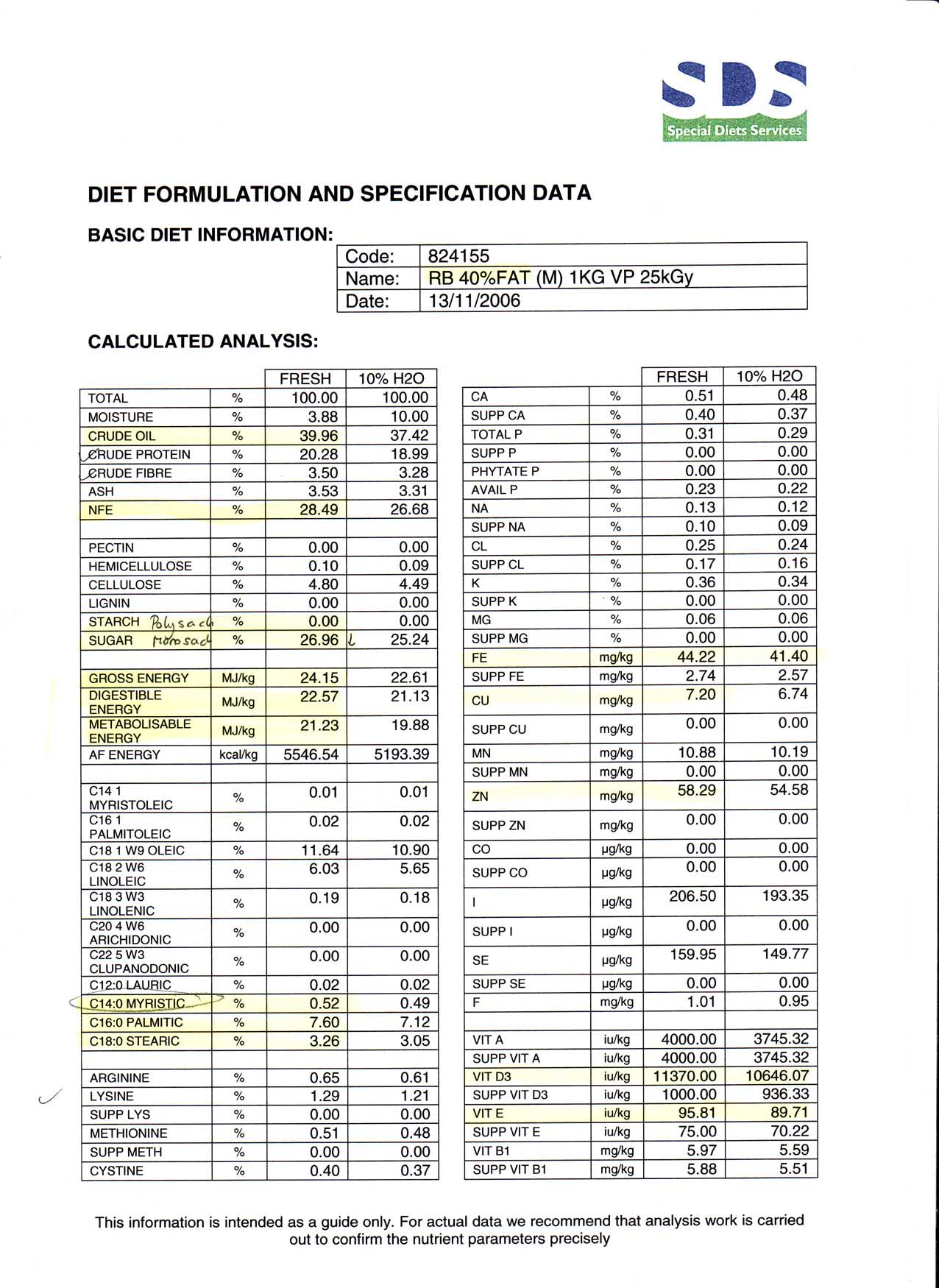

Supplement: Table S1 — Composition of the high fat diet used to feed C57BL/6J and BALB/c mice. (DOCX) [file pone.0082825.s001.docx]
