# Supplementary material for: Nutrigenomics of High Fat Diet Induced Obesity in Mice Suggests Relationships between Susceptibility to Fatty Liver Disease and the Proteasome
Source: PLoS One. 2013 Dec 6;8(12):e82825. doi: 10.1371/journal.pone.0082825 (PMC3855786; doi:10.1371/journal.pone.0082825)
Supplement: Table S2 — Oligonucleotides designed for quantitative RT-PCR analysis of gene transcription regulation in C57BL/6J and BALB/c mice. (DOCX) [file pone.0082825.s002.docx]

**Table S2.** Oligonucleotides designed for quantitative RT-PCR analysis of gene transcription regulation in C57BL/6J and BALB/c mice.

Gene Forward primer (5-'3') Reverse primer (5-'3')

Gapdh AGGTCGGTGTGAACGGAT TGTAGACCATGTAGTTGAGG

Pparg GCGATCTTGACAGGAAAGA GGGTGATATGTTTGAACTTGA

Psma5 AGTACGACAGGGGTGTGA TCTTCTCCACAGCTAGACAT

Psmc4 ATTCCAGCACTGTCCGTG GATATACTCCTCCTGCACTT

Psmb9 TCACTCTGGCCATGAACC GTCACTCATCGTAGAATTTTG

Psmd2 GTTCCTGGAGCTGAGTGA TAGATGTCATCAGGCACCT

Rbx1 ATGGATGTGGATACCCCC TGTGGTTCCTGCAGATGG

Anapc2 ATAGAGAGAGTGGTTGGTTG GCATAGATACGGTAGAAGAAT
