# Supplementary material for: Nutrigenomics of High Fat Diet Induced Obesity in Mice Suggests Relationships between Susceptibility to Fatty Liver Disease and the Proteasome
Source: PLoS One. 2013 Dec 6;8(12):e82825. doi: 10.1371/journal.pone.0082825 (PMC3855786; doi:10.1371/journal.pone.0082825)

Figure S1. GSEA-derived enrichment plots for the JAK-STAT signalling pathway in the BALB/c (A) and C57BL6/J (B) mice, showing HFD-induced expression upregulation of the vast majority of genes in this pathway in BALBL/c (A) and an equivalent proportion of genes up- and down regulated in C57BL6/J (B).

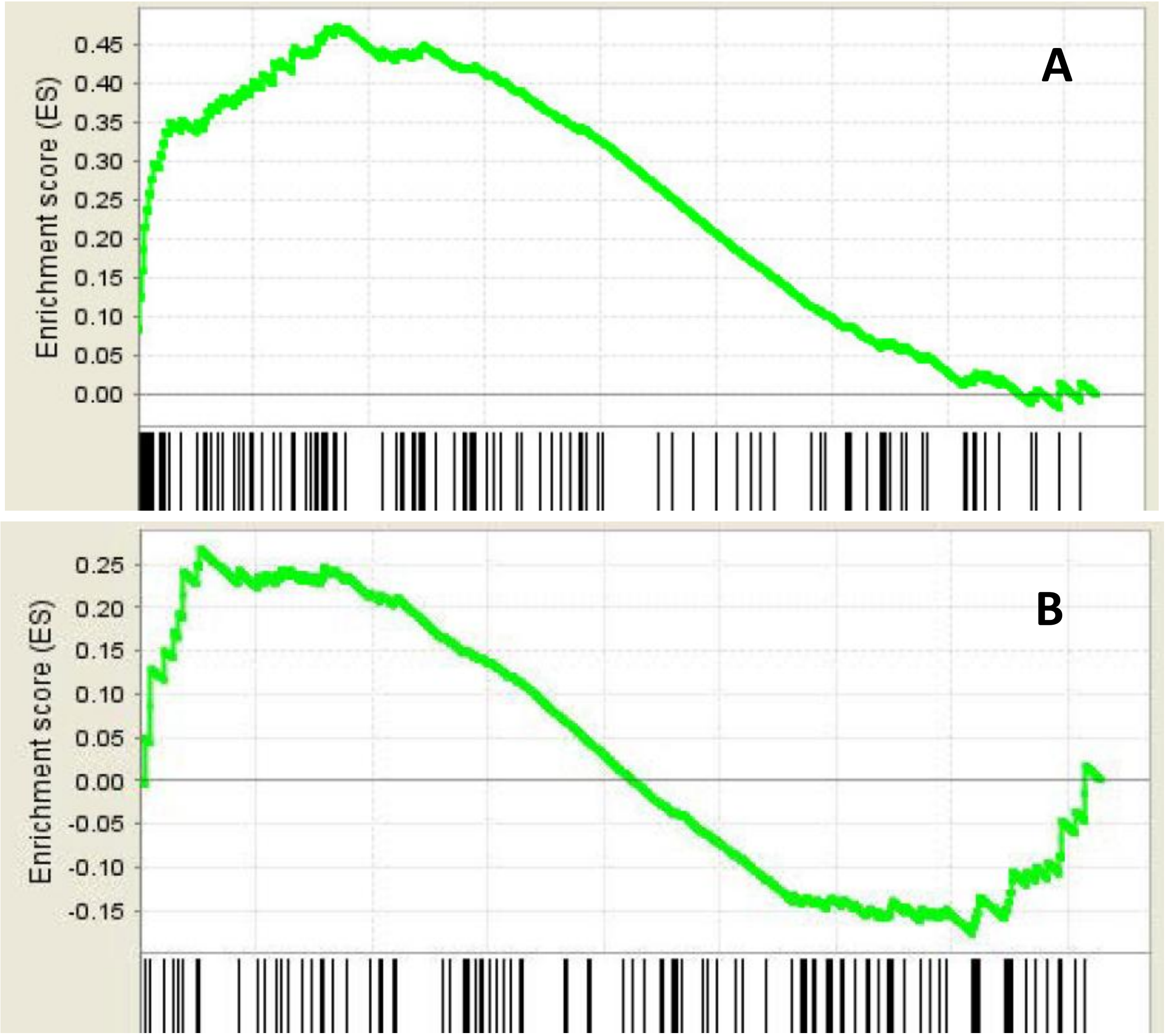

Supplement: Figure S1 — GSEA-derived enrichment plots for the JAK-STAT signalling pathway in BALB/c and C57BL6/J mice. (PDF) [file pone.0082825.s005.pdf]
