# Supplementary material for: Nutrigenomics of High Fat Diet Induced Obesity in Mice Suggests Relationships between Susceptibility to Fatty Liver Disease and the Proteasome
Source: PLoS One. 2013 Dec 6;8(12):e82825. doi: 10.1371/journal.pone.0082825 (PMC3855786; doi:10.1371/journal.pone.0082825)

Figure S2. Pathway analysis of the effects of HFD on liver gene transcription in C57BL/6J and 129S6 mice. Only KEGG biological pathways identified by GSEA as significantly affected by HFD in at least one of the strains are reported. False discovery rate (FDR) q-values (<0.05) were used to test for statistically significant pathway upregulation (red) or downregulation (green) in response to fat feeding in 129S6 (*) and C57BL/6J ($) mice.

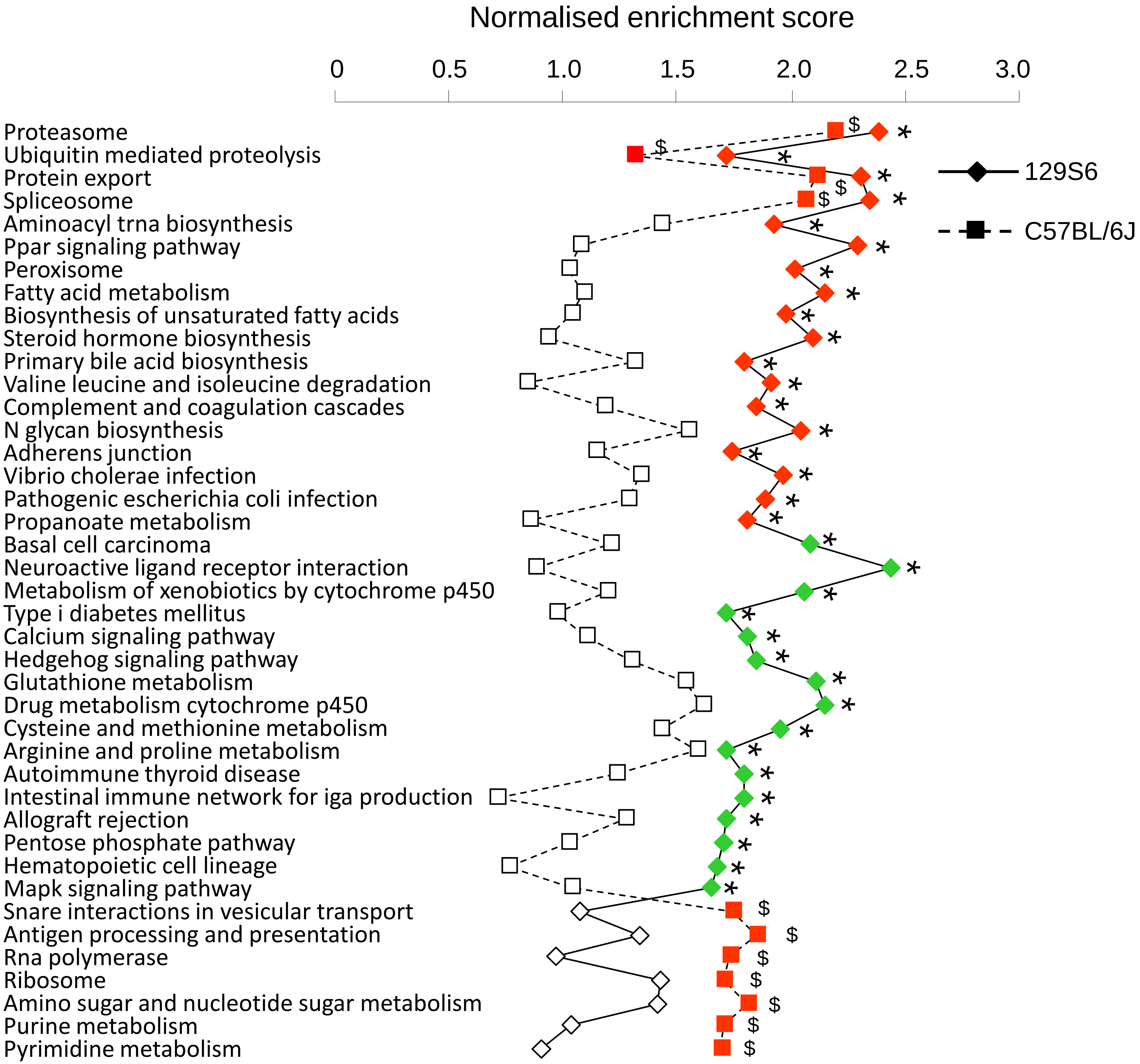

Supplement: Figure S2 — Pathway analysis of the effects of high fat diet on liver gene transcription in C57BL/6J and 129S6 mice. (PDF) [file pone.0082825.s006.pdf]
